# Proton Beam Therapy in Combination with Intra-Arterial Infusion Chemotherapy for T4 Squamous Cell Carcinoma of the Maxillary Gingiva

**DOI:** 10.3390/cancers10090333

**Published:** 2018-09-15

**Authors:** Hiromasa Endo, Kanako Takayama, Kenji Mitsudo, Tatsuya Nakamura, Ichiro Seto, Hisashi Yamaguchi, Takashi Ono, Motohisa Suzuki, Yusuke Azami, Hitoshi Wada, Masao Murakami, Iwai Tohnai

**Affiliations:** 1 Department of Radiation Oncology, Southern Tohoku Proton Therapy Center, 7-172, Yatsuyamada, Koriyama, Fukushima 963-8052, Japan; asamori612@gmail.com (H.E.); tatsuya.nakamura@nifty.com (T.N.); setoichiro@gmail.com (I.S.); sisasimaru@ezweb.ne.jp (H.Y.); abc1123513@gmail.com (T.O.); asihotom.lassen@gmail.com (M.S.); azamiysuke@gmail.com (Y.A.); hhktk981@gmail.com (H.W.); mmuraka2013@gmail.com (M.M.); 2 Department of Oral and Maxillofacial Surgery, Yokohama City University Graduate School of Medicine, 3-9, Fukuura, Kanazawa, Yokohama, Kanagawa 236-0004, Japan; mitsudo@yokohama-cu.ac.jp (K.M.); tohnai@yokohama-cu.ac.jp (I.T.)

**Keywords:** maxillary gingival cancer, proton beam therapy, intra-arterial infusion chemotherapy, chemoradiotherapy, oral squamous cell carcinomas

## Abstract

This study aimed to evaluate the therapeutic effect and toxicity of proton beam therapy in combination with intra-arterial infusion chemotherapy in patients with squamous cell carcinoma of the maxillary gingiva. Between December 2010 and March 2016, 30 patients with T4 squamous cell carcinoma of the maxillary gingiva were treated with radiotherapy and retrograde intra-arterial infusion chemotherapy using cisplatin (20–40 mg/m^2^, 4–6 times). Radiotherapy was basically administered using boost proton beam therapy for primary tumor and neck lymph node tumors, following 36–40 Gy photon radiation therapy delivered to the prophylactic area, to a total dose of 70.4–74.8 Gy. The median follow-up was 33 months. The 3-year local control and overall survival rates were 69% and 59%, respectively. Major grade 3 or higher acute toxicities included mucositis, neutropenia, and dermatitis in 12 (40%), 5 (17%), and 3 (10%) patients, respectively. No grade 3 or higher late toxicities were observed. These results suggested that proton beam therapy in combination with intra-arterial infusion chemotherapy was not inferior to other treatment protocols and should be considered as a safe and effective option in patients with T4 squamous cell carcinoma of the maxillary gingiva.

## 1. Introduction

Malignant tumors of the maxillary gingiva are rare, representing approximately 5% of all head and neck malignancies, and approximately 90% of the cases in Japan are squamous cell carcinoma (SCC) [1]. Standard treatment for oral SCC is surgery with or without chemotherapy and postoperative radiotherapy [2,3,4]. Surgical approaches for locally advanced maxillary gingival cancer, including maxillary partial resection, total maxillectomy, and craniofacial resection with or without orbital exenteration, result in significant disfigurement and functional impairment.

Nearly all T4b maxillary gingival tumors are unresectable due to the involvement of the clivus, middle cranial fossa, and brain as well as the invasion to the pterygoid process and the masticatory space. Despite the frequent use of definitive chemoradiotherapy in T4b tumors, their therapeutic effect is not favorable.

In recent decades, intra-arterial infusion chemoradiotherapy has been performed for locally advanced head and neck cancers to avoid extended surgery. Several studies reported that the results with this organ-preserving approach were not inferior to those with surgery [5,6,7,8]. However, late adverse events after radiation, such as osteoradionecrosis and dry mouth, cannot be ignored [9,10,11].

Proton beam has the major advantage of a rapid dose fall-off at the distal end of the Bragg peak [12], which underlies the better dose distribution with proton beam therapy (PBT) compared with conventional X-ray therapy (XRT). And also has possibility to induce double-strand breaks within the double helical structure of DNA and cause catastrophic damage to cancer cells [13,14]. PBT is an effective approach that provides curative, high-dose irradiation to the tumor volume without increasing normal tissue toxicity. PBT appears to be suitable for treating head and neck tumors with complex anatomy. However, few studies reported on the outcomes of PBT in oral SCC [15,16], and no reports to date investigated its efficacy in SCC of the maxillary gingiva.

The aim of this study was to evaluate the therapeutic effects and outcomes of PBT with intra-arterial infusion chemotherapy (IACT) in patients with T4 SCC of the maxillary gingiva.

## 2. Results

### 2.1. Patient Characteristics

Thirty patients with T4 SCC of the maxillary gingiva were treated with PBT and IACT between December 2010 and March 2016. Patient characteristics are summarized in Table 1. The median age was 68 (range, 50–86) years, and there were 16 males (53%) and 14 females (47%). 

Of the 30 patients, 10 (33%) and 20 (67%) patients were diagnosed with T4a and T4b SCC, respectively. Twenty-one patients (70%) had cervical lymph node metastasis at the treatment initiation, and 18 patients (60%) had unresectable disease. Four of the twelve patients with resectable disease were inoperable because of old age, whereas the remaining eight patients refused surgery.

### 2.2. Compliance

All patients completed PBT and at least four cycles of IACT (median: 6 (range, 4–11)). The median total cisplatin dose of all patients was 290 (range, 180–485) mg/body, and the median total radiation dose of all patients was 70.8 (range, 57–74.8) Gy for the primary tumor site, and 70.8 (range, 57–79.6) Gy for the metastatic cervical lymph nodes, respectively. The irradiation boost fields were prepared separately for the primary lesion and the cervical lymph nodes, and the same dose was used in patients with overlapping irradiation fields. The median prohpylactic X-ray dose for cervical lymph nodes was 40 Gy in 20 fractions (range, 26–40 Gy). The median proton beam dose was 33 Gy (relative biological effectiveness (RBE)) in 15 fractions (RBE range, 26.4–44 Gy) for the primary tumor site and 33 Gy (RBE) in 15 fractions (RBE range, 26.4–39.6 Gy) for the metastatic cervical lymph nodes. Four patients were treated with PBT only, in whom the median proton beam dose was 72.6 Gy (RBE) in 33 fractions (range 70.4–74.8 Gy (RBE)).

### 2.3. Response and Survival

Twenty-six of the thirty patients (87%) achieved complete response (CR), and four patients (13%) achieved partial response (PR) in the primary lesion. The median follow-up duration was 33 (range, 5–73) months. During the observation period, local recurrence or remnant in the primary site were observed in nine patients. Three of these patients underwent salvage surgery, and tumor control was achieved in one patient; the remaining six patients were inoperable, because of the close proximity of the tumors to the skull base or the internal carotid artery in four patients, distant metastasis in one patient, and refusal of surgery in one patient. Tumor recurrence in regional lymph nodes occurred in four patients, all of whom underwent additional radiotherapy and achieved locoregional control; however, distant metastases occurred in two of these patients. Consequently, 12 patients (40%) died due to the following causes: progression of the primary lesion (*n* = 5, 17%), distant metastasis (*n* = 5, 17%), and non-cancer-related causes (cerebral infarction and pneumonia; *n* = 2, 6.7%).

**Figure 1:**
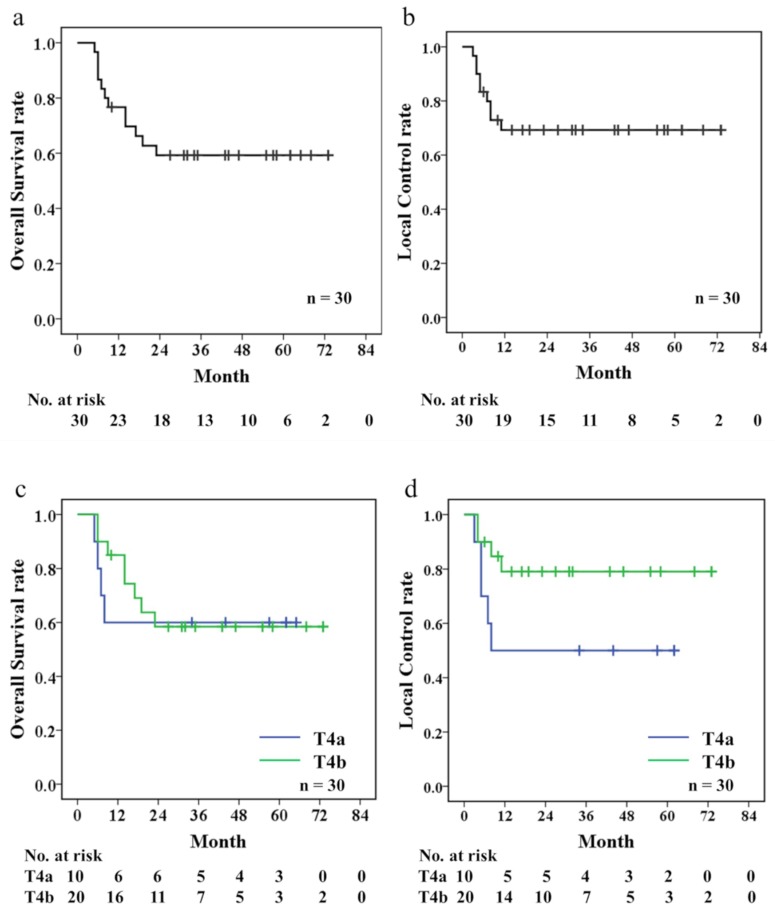
Kaplan-Meier estimates of Overall survival (OS) (**a**), local control (LC) (**b**), Comparisons bet ween T4a and T4b patients to OS (**c**), and LC (**d**).

The 3-year local control (LC) and overall survival (OS) rates were 69% and 59%, respectively. Additionally, the 3-year LC and OS rates in the T4a subgroup were 50% and 60%, while those in the T4b subgroup group were 79% and 58%, respectively (Figure 1). No significant differences in the LC or OS rates were observed between the patients with T4a and T4b SCC (*p* = 0.185 and *p* = 0.918, respectively).

The following factors were evaluated for their potential involvement with OS and LC: age (<70 years vs. ≥70 years), sex (male vs. female), surgical indication (operable vs. inoperable), tumor size (<5.0 cm vs. ≥5.0 cm), and total radiation dose (<70 Gy vs. ≥70 Gy). Tumor size had a significant association with both OS and LC (Table 2). However, as other factors did not exhibit a significant association, multivariate analysis was not performed.

### 2.4. Toxicities

Table 3 shows acute and late toxicities that were observed during the treatment and follow-up periods. No grade 5 toxicities were observed in the study cohort, and the grade 4 toxicity of neutropenia was observed in only one patient (3.3%). Conversely, grade 3 toxicities included mucositis, neutropenia, dermatitis, and thrombocytopenia in 12 (40%), 4 (13%), 3 (10%), and 1 (3.3%) patient, respectively. There were no cerebrovascular disorders, and no grade 3 or higher late toxicities were observed. Grade 2 or lower late toxicities included dry mouth, dysgeusia, osteonecrosis, cataract, middle ear inflammation, and optic nerve disorder in 22 (73%), 7 (23%), 4 (13%), 2 (6.7%), 2 (6.7%), and 1 (3.3%) patient, respectively. None of the patients developed brain necrosis, olfactory nerve disorder, retinopathy, or dysphagia.

## 3. Discussion

In the present study including 30 patients treated with PBT and IACT; the 3-year OS and LC rates were 59% and 69%, respectively. Previous studies reported that the 5-year OS rates after initial surgical treatment for all stages of SCC of the hard palate and maxillary alveolus ranged from 32 to 44% [4,17,18]. Lin et al. reported a 5-year OS rate of 26.4% in patients with T4 SCC of the hard palate and maxillary alveolus who were treated with initial surgery [3]. Several studies investigated the outcomes of radical concurrent chemoradiotherapy (CCRT) for SCC of the maxillary gingiva. Specifically, Wang et al. reported that the 3-year OS rate in patients with T4 and T3N3 SCC of the hard palate and maxillary alveolus who were treated with CCRT including intravenous chemotherapy with 70-Gy XRT was only 15% [19]. In the present study, the 3-year OS rate was 59%, although all patients were T4, with the majority diagnosed with inoperable disease. These findings provide evidence that PBT in combination with IACT might be equal to or better than conventional treatments.

Of the 26 patients who achieved CR, 5 (19%) had local recurrence, whereas all four patients who achieved PR (100%) relapsed during the follow-up period. These local recurrences occurred within the first two years in the current study. Increasing the prescribed dose of radiation therapy, which might improve LC, increases the risk of toxicities. Conversely, the combined use of IACT and radiation therapy might contribute to the improvement of the LC. We utilized IACT instead of intravenous chemotherapy in the current study. IACT delivers a high concentration of anticancer drugs to the tumor sites and has the potential to increase their therapeutic effects. There are currently two IACT approaches for head and neck cancers: one that utilizes a catheter inserted into the target artery through the superficial temporal artery (STA) as presented in the current study, and one that utilizes a catheter inserted into the target artery through the femoral artery using the Seldinger method. The latter method can sometimes cause serious neurological complications [20,21]. In the present study, catheterization was retrogradely performed through the STA, and no neurological complications were observed. This method is safe in long-term repeated IACT for head and neck cancers. Three-dimensional computed tomography angiography (3D-CTA) played an important role in the treatment strategy of catheter infusion by identification of the feeding artery of the tumor. Beneficial treatment outcomes of IACT in combination with XRT were previously reported in patients with maxillary gingival SCCs [8,22]. Mitsudo et al. reported the clinical outcomes of treatment with daily IACT + conventional XRT in a series of 112 patients with stage III and IV oral SCC, including 16 patients with maxillary gingival cancer [8]. Although the 5-year LC and OS were 79.3% and 71.3%, respectively, they included cases in which neck dissection was performed. Many patients with maxillary gingival carcinoma develop cervical lymph node metastases, unlike those with maxillary sinus carcinoma. Furthermore, patients with tumors that extend across the midline to the contralateral side tend to develop lymph node metastases on both sides. The standard treatment for lymph node metastasis in the neck is neck dissection. In the present study, 12 patients (40%) had cervical lymph node metastases on the affected side and 9 patients (30%) had bilateral cervical lymph node metastases. The status for cervical lymph node metastasis was well controlled in the current study, which was an important advantage of our analysis. The 3-year LC and OS rates in the current study suggest PBT in combination with IACT as a beneficial treatment approach in maxillary gingival SCC, given that all study patients had T4 disease. Furthermore, no significant differences in the LC and OS rates were observed between those with T4a and T4b disease, suggesting that combination chemotherapy with PBT and IACT might provide curative treatment for patients with inoperable T4b diagnosis as well as those with T4a disease. T4a tumor was rather worse than T4b in LC. The bone invasion of the tumor and related bone destruction were found to be more advanced in the patients with T4a tumors than in those with T4b tumors. It is possible that efficient anticancer drug concentration was not achieved because of poor blood flow in the bone in the patients with severe bone destruction. Furthermore, the univariate analysis which revealed that a primary tumor size smaller than 5.0 cm was associated with significantly better LC and OS suggested that the tumor size might play a more important role in LC and OS than the extent of tumor progression. Some studies on oral cancer have reported that tumor volume is not a clear prognostic factor in surgical cases, but it is an important prognostic factor in chemoradiotherapy [23,24]. In the present study, we found a significant difference in the tumor size, which bolstered our theory that tumor size was an important prognostic factor in chemoradiotherapy.

The most frequent acute toxicity in the current study was oral mucositis. Previous studies on IACT in combination with XRT reported that, despite severe acute toxicities, grade 3 or 4 oral mucositis was observed in 50–86% of patients and that few systemic toxicities were also noted [8,22]. In IACT, systemic toxicities are reduced using sodium thiosulfate (STS) in combination with intra-arterial cisplatin infusion [21]. In addition, in the current study, PBT boost was used to reduce toxicities. Van de Water et al. reported that PBT delivered lower doses to the organs at risk and improved target dose coverage and conformity [25]. PBT can deliver high-dose of radiation to the tumor site, while minimizing the doses delivered to the surrounding normal tissues. Compared to that observed with conventional photon therapy, the frequency of oral mucositis was lower in the current study using PBT. There are no reports of PBT-associated toxicities during treatment of SCC of the maxillary gingiva and alveolus; however, several studies on PBT for SCC of the nasal cavity and paranasal sinuses reported that the frequencies of grade 3 or higher optic nerve disorders or cataracts ranged from 5.6% to 29% [26,27]. In the present study, despite the two patients with orbital invasion of the tumor and six patients with orbital floor invasion close to the tumor, there were no grade 3 or higher optic nerve disorders or cataracts after treatment. A good dose distribution of the proton beams might have reduced acute and late toxicities.

We acknowledge that there are limitations to this retrospective study with a small patient population, which therefore has inherent biases. We attempted to overcome these limitations by including a homogeneous study population of patients with only T4 SCCs. The follow-up period was short; therefore, the follow-up of the study cohort is ongoing to further investigate the long-term prognosis and late toxicities.

## 4. Materials and Methods 

### 4.1. Patients and Study Design

**Figure 2:**
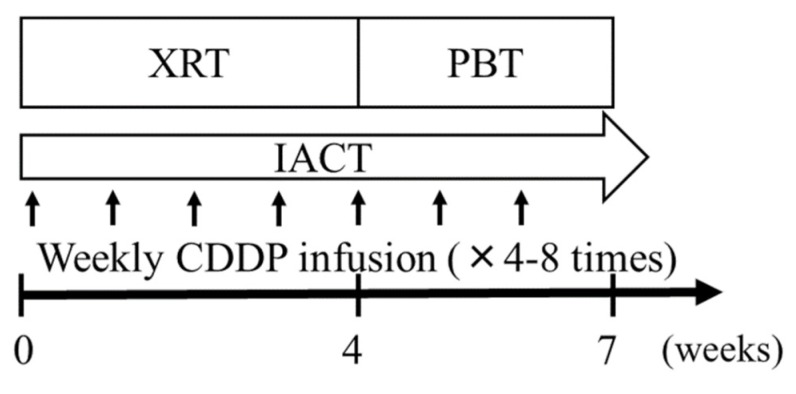
Treatment schedule. XRT, X-ray therapy (36–40 Gy in 20 fractions); PBT, Proton beam therapy (28.6–33.0 Gy in 13–15 fractions); IACT, intra-arterial infusion chemotherapy; CDDP, cisplatin (20–40 mg/m^2^).

The eligibility criteria were as follows: (1) pathologically confirmed SCC of the maxillary gingiva; (2) no distant metastasis; (3) no history of radiotherapy or surgery for a head and neck cancer; (4) Eastern Cooperative Oncology Group performance score, 0–2; (5) adequate hematological parameters (white blood cell count, >3500/μL; neutrophil count, >2000/μL; platelet count, >1 × 10^6^/μL; hemoglobin, >9 g/dL); (6) normal hepatic and renal function; and (7) follow-up duration >12 months. Disease evaluation included physical examination, chest radiography, CT, magnetic resonance imaging (MRI), and fluoro-2-deoxy-D-glucose positron emission tomography-CT (PET-CT). Tumor staging was based on the Union for International Cancer Control TNM Classification of Malignant Tumors, 7th edition [28]. Given that all cases were T4 tumors and that there were no obvious extranodal extensions/infiltrations, the differences between the 7th and 8th editions of the TNM classification, which do not extend to T4 tumors, did not affect the patient staging. Treatment schedules are summarized in Figure 2. All patients received CCRT, which comprised daily and weekly IACT. Written informed consent was obtained from all subjects prior to the initiation of treatment. This study was approved by the ethics committee of the study institute (IRB number D17-26, 18 July 2017). This study was conducted according to the principles of the Declaration of Helsinki.

### 4.2. Radiotherapy

The patients were treated using external irradiation. Since the PBT system at the study institution (Mitsubishi Electric Corporation, Tokyo, Japan) was not suitable to deliver radiation over a wide dose range, prophylactic irradiation of the regional lymph nodes was performed using XRT, including the regional lymph nodes on either the affected side (N0 or N1) or both sides (>N2) [7]. The radiation dose for XRT was 36–40 Gy in 20 fractions, which was performed five times a week, using 6 MV X-rays (Clinac iX, Varian Medical Systems, Tokyo, Japan). The irradiation field contained the primary tumor and cervical lymph node areas and depended on the extent of metastasis.

For PBT planning, we developed CT-based 3-dimensional treatment planning system (Xio-M, ELEKTA, Stockholm, Sweden). CT images were overlapped with enhanced MRI images. To determine gross tumor volume (GTV), we focused only on the primary tumor and regional lymph node metastases. Clinical target volume (CTV) was defined as the GTV with a 3–5-mm margin in all directions, and planning target volume (PTV) was defined as the CTV plus a 3-mm margin. The proton beam dose was defined as the physical dose multiplied by a relative biological effectiveness (RBE) value of 1.1 and described in units of Gy (RBE). The additional PBT boost dose after XRT was 28.6–33.0 Gy (RBE) in 13–15 fractions, with 2.2 Gy (RBE) per fraction. Patients who were classified as N0 and old age (> 70 years old) were treated with PBT only and did not receive prophylactic cervical irradiation. In these patients, the total PBT dose was 70.4–74.8 Gy (RBE) in 32–34 fractions, with 2.2 Gy (RBE) per fraction. Irradiation was performed five times a week using 150 or 210 MeV proton beams, with the dual-portal broad-beam method and multi-leaf collimators. The patients were positioned and immobilized with an oral spacer and thermoplastic head mask shell to ensure high repositioning accuracy of the target. The spacer was used to avoid extra irradiation to the tongue and the mandible.

### 4.3. Intra-Arterial Infusion Chemotherapy

**Figure 3:**
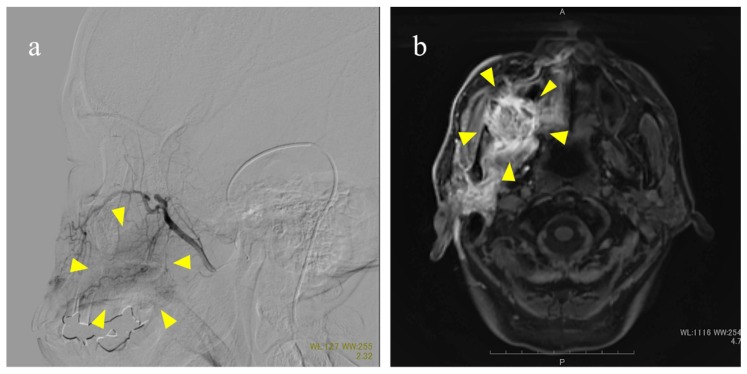
Methods to confirm flow using digital subtraction angiography (**a**) and magnetic resonance imaging (**b**). (**a**) The maxillary artery is displayed. Arrows indicate the labeled maxillary gingival tumor; (**b**) Perfusion of the right maxillary gingiva, palate, pterygoid muscle, and parotid gland. Arrows indicate the enhanced tumor. Scale bar: 10mm.

Prior to treatment, 3D-CTA of the carotid artery was performed to identify the arteries feeding the tumor site and determine the morphology and the course of the feeding arteries extending from the external carotid artery. Catheterization through the STA was performed as previously reported [29]. Briefly, an incision was made anterior to the ear on the affected side under local anesthesia to expose the STA. Under fluoroscopy, the tip of the catheter was placed into the external carotid artery. Maxillary gingival and alveolar cancers are typically surrounded by the region that is supplied by the maxillary artery; however, the facial artery, transverse facial artery, ascending pharyngeal artery, and innominate arteries branching from the main external carotid artery can sometimes be involved. To target the entire tumor spreading the surrounding tissue, therefore, the tip of the catheter was placed in a location where the anticancer agents were expected to flux through the entire tumor. Considering the blood flow, the catheter tip was selectively placed into the facial artery, if necessary. If the primary tumor extended beyond the median line into the contralateral side, another catheter was inserted into the contralateral side for bilateral arterial injection. The extent of the arterial injection was confirmed by injecting an extremely low-dose contrast medium into the tumor with an arterial injection catheter and evaluating with digital subtraction angiography and MRI [30] (Figure 3). 

Cisplatin was administered at 20–40 mg/m^2^ over five hours, once a week, for a total of four to six times. When the catheter was inserted selectively, the dose of cisplatin was set at up to 20 mg/m^2^. During intra-arterial infusion of cisplatin, the cisplatin-neutralizing agent STS was also administered intravenously at 8 g/m^2^ over eight hours, starting one hour before the infusion of cisplatin. The dose of STS was set in accordance with the dose of cisplatin. A 5-hydroxytryptamine 3 (5-HT 3) receptor antagonist and corticosteroids were administered to minimize nausea and vomiting in all patients before intra-arterial infusion.

### 4.4. Follow-up Evaluation and Analysis

Clinical response was evaluated based on the results of physical examination and an enhanced MRI, which were performed four weeks after treatment, according to the Response Evaluation Criteria in Solid Tumors guidelines, version 1.1 [31]. All patients were observed every three months for 3 years after therapy and every 6 months thereafter. Acute and late toxicities were graded according to the Common Terminology Criteria for Adverse Events, version 4.0 [32].

OS was calculated from the last day of PBT to the date of death or last confirmed date of survival. LC was calculated from the last day of PBT to the date of local recurrence. The OS and LC curves were estimated using the Kaplan–Meier method, and the log-rank test was used for univariate analysis including age, sex, primary tumor size, surgical indication, and total radiation dose as independent variables. All *p* values were two-sided, and a *p* value <0.05 was considered to indicate statistical significance. All statistical analyses were performed using International Business Machines Corporation (IBM) SPSS statistics version 22 (SPSS, Chicago, IL, USA).

## 5. Conclusions

PBT in combination with IACT is a safe and effective treatment for T4 SCC of the maxillary gingiva and should be considered as a viable treatment option for T4 maxillary gingival cancer.

## Figures and Tables

**Table 1:** Patient and treatment characteristics.

Characteristics	*n* or Median (% or Range)
Number of patients	30
Age (years); median	68 (50–86)
Gender	
Male	16 (53)
Female	14 (47)
ECOG-PS	
0	21 (70)
1	6 (20)
2	3 (10)
T Classification ^1^	
T4a	10 (33)
T4b	20 (67)
N Classification ^1^	
N0	9 (30)
N1	4 (13)
N2b	8 (27)
N2c	9 (30)
Tumor size; median (mm)	54 (27–78)
Reasons for not performing surgery	
operable; refusal	8 (27)
old age	4 (13)
inoperable; advanced	15 (50)
other reason	3 (10)

^1^ UICC, Union for International Cancer Control TNM classification, 7th edition; Abbreviations: EOCG-PS, Eastern Cooperative Oncology Group Performance status.

**Table 2:** Results of log-rank tests for prognostic factors.

Factors	No. of Patients	*p* Value	
		OS	LC
Age		0.813	0.537
<70 years	17		
≥70 years	13		
Sex		0.300	0.361
Male	16		
Female	14		
Surgical indication		0.058	0.196
operable	12		
inoperable	18		
Tumor size		0.049	0.048
<5.0 cm	12		
≥5.0 cm	18		
Total radiation dose		0.782	0.571
<70 Gy	8		
≥70 Gy	22		

**Table 3:** Adverse events (NCI-CTCAE v.4.0) ^1^.

Toxicity	Grade, *n* (%)			
	1	2	3	4
Acute				
Mucositis Oral	0	18 (60)	12 (40)	0
Dermatitis	18 (60)	9 (30)	3 (10)	0
Neutropenia	12 (40)	7 (23)	4 (14)	1 (3)
Anemia	10 (33)	12 (40)	0	0
Platelet count decreased	1 (3)	1 (3)	1 (3)	0
Nausea	7 (23)	5 (17)	1 (3)	－
Acute kidney injury	1 (3)	0	0	0
Hepatobiliary disorders	0	0	0	0
Fever	12 (40)	2 (7)	0	0
Late				
Dry mouth	14 (47)	8 (27)	0	－
Dysgeusia	7 (23)	1 (3)	－	－
Osteonecrosis of jaw	2 (7)	2 (7)	0	0
Cataract	0	2 (7)	0	0
Optic nerve disorder	0	1 (3)	0	0
Middle ear inflammation	1 (3)	1 (3)	0	0

^1^ NCI-CTCAE v.4.0, National Cancer Institute-Common Terminology Criteria for Adverse Events, version 4.0. *n*: number
